# Screening HIV-Infected Patients with Low CD4 Counts for Cryptococcal Antigenemia prior to Initiation of Antiretroviral Therapy: Cost Effectiveness of Alternative Screening Strategies in South Africa

**DOI:** 10.1371/journal.pone.0158986

**Published:** 2016-07-08

**Authors:** Bruce A. Larson, Peter C. Rockers, Rachael Bonawitz, Charlotte Sriruttan, Deborah K. Glencross, Naseem Cassim, Lindi M. Coetzee, Gregory S. Greene, Tom M. Chiller, Snigdha Vallabhaneni, Lawrence Long, Craig van Rensburg, Nelesh P. Govender

**Affiliations:** 1 Department of Global Health, Boston University School of Public Health, Boston, Massachusetts, United States of America; 2 National Institute for Communicable Diseases – Centre for Opportunistic, Tropical and Hospital Infections, Johannesburg, South Africa; 3 National Health Laboratory Service, Johannesburg, South Africa; 4 Faculty of Health Sciences, University of the Witwatersrand, Johannesburg, South Africa; 5 Health Economics and Epidemiology Office, Wits Health Consortium, Johannesburg, South Africa; 6 Mycotic Diseases Branch, Centers for Disease Control and Prevention, Atlanta, Georgia, United States of America; 7 Department of Pediatrics, Boston University School of Medicine, Boston, Massachusetts, United States of America; Faculty of Medicine, AUSTRALIA

## Abstract

**Background:**

In 2015 South Africa established a national cryptococcal antigenemia (CrAg) screening policy targeted at HIV-infected patients with CD4+ T-lymphocyte (CD4) counts <100 cells/ μl who are not yet on antiretroviral treatment (ART). Two screening strategies are included in national guidelines: reflex screening, where a CrAg test is performed on remnant blood samples from CD4 testing; and provider-initiated screening, where providers order a CrAg test after a patient returns for CD4 test results. The objective of this study was to compare costs and effectiveness of these two screening strategies.

**Methods:**

We developed a decision analytic model to compare reflex and provider-initiated screening in terms of programmatic and health outcomes (number screened, number identified for preemptive treatment, lives saved, and discounted years of life saved) and screening and treatment costs (2015 USD). We estimated a base case with prevalence and other parameters based on data collected during CrAg screening pilot projects integrated into routine HIV care in Gauteng, Free State, and Western Cape Provinces. We conducted sensitivity analyses to explore how results change with underlying parameter assumptions.

**Results:**

In the base case, for each 100,000 CD4 tests, the reflex strategy compared to the provider-initiated strategy has higher screening costs ($37,536 higher) but lower treatment costs ($55,165 lower), so overall costs of screening and treatment are $17,629 less with the reflex strategy. The reflex strategy saves more lives (30 lives, 647 additional years of life saved). Sensitivity analyses suggest that reflex screening dominates provider-initiated screening (lower total costs and more lives saved) or saves additional lives for small additional costs (< $125 per life year) across a wide range of conditions (CrAg prevalence, patient and provider behavior, patient survival without treatment, and effectiveness of preemptive fluconazole treatment).

**Conclusions:**

In countries with substantial numbers of people with untreated, advanced HIV disease such as South Africa, CrAg screening before initiation of ART has the potential to reduce cryptococcal meningitis and save lives. Reflex screening compared to provider-initiated screening saves more lives and is likely to be cost saving or have low additional costs per additional year of life saved.

## Background

Cryptococcal meningitis (CM) is a leading cause of death among HIV-infected patients with low CD4+ T-lymphocyte (CD4) counts. Further, patients with undiagnosed cryptococcal disease at time of antiretroviral treatment (ART) initiation are at high risk for death from immune reconstitution inflammatory syndrome (IRIS) [1–3]. Cryptococcal antigenemia (CrAg) can be diagnosed weeks before CM onset with near-perfect sensitivity and specificity [4, 5], and treatment of CrAg positive patients who do not already have CM with high-dose fluconazole reduces both progression to CM and the risks of death from cryptococcal IRIS [6–8]. The median time between becoming CrAg positive and developing CM is three weeks [5], making it imperative to identify and treat CrAg positive patients quickly. Previous research suggests that, compared to no screening, a CrAg screen-and-treat approach prior to the initiation of ART among patients with low CD4 counts is cost effective [9–12].

As of 2015, the South African government recommended that “HIV-positive adults with a CD4 count <100 cells/μl should be screened for cryptococcal disease before ART is started” [13]. The relevant issue is how to most efficiently integrate CrAg screening into a large HIV treatment program. Two CrAg screening strategies are currently included in South African guidelines: reflex and provider-initiated screening (see page 99 in [13]). With reflex screening, a single patient blood sample is drawn at the time of HIV diagnosis for a baseline CD4 count, the current standard of care. The lab tests all remnant samples with CD4 counts < 100 cells/μl for CrAg (a qualitative result showing positive or negative), regardless of patient's ART or prior CM status, because both are unknown to lab staff. Finally, providers (generally nurses), review both CD4 and CrAg results when patients return for their second visit. With provider-initiated screening, a blood sample is drawn at the time of HIV diagnosis for an initial CD4 test, followed by review of CD4 results during a second patient visit to the clinic. At this second visit, providers must determine if a CrAg test is indicated (i.e. CD4 count < 100 cells/μl, ART naïve, and no prior CM), draw a second blood sample, and send it to the lab. The patient must then return for a third visit to review the results of the CrAg test.

In this paper, we present a decision-analytic model to compare reflex and provider-initiated CrAg screening strategies based on costs (2015 USD), disaggregated into screening, preemptive treatment to avoid hospitalization, hospital, and post-hospital costs, and health outcomes (lives saved and years of life saved using a 3% discount rate). Although prior research has shown that CrAg screening prior to ART initiation is cost effective, alternative strategies for CrAg screening have not been compared systematically. Thus, the focus here is on the difference in costs between the two strategies, the difference in health outcomes achieved, and the incremental cost effectiveness of reflex compared to provider-initiated screening.

Of particular interest is the impact of provider and patient adherence to the different strategy protocols. The provider-initiated strategy places responsibility on providers to assess correctly patient eligibility for CrAg screening, while the reflex strategy does not. Both strategies require patients to return for a second visit. High rates of loss-to-follow-up after HIV diagnosis are well documented in South Africa and elsewhere [14–18], which affects both screening strategies. The provider-initiated policy additionally requires a patient to return for a third visit to obtain CrAg results, which creates another opportunity for patients to be missed by this screening strategy. In addition, because a third visit would be required prior to ART initiation with a provider-initiated policy, ART initiation could be delayed for the majority of targeted patients (pre-ART, CD4 count < 100 cells/μl) who will screen CrAg negative. While not discussed further in this paper, potential ethical differences between the two strategies could also be considered, since patients with CD4 counts < 100 cells/μl by definition have advanced HIV disease, and provider-initiated screening involves more delay in treatment, either initiation of ART for those CrAg negative or fluconazole for those CrAg positive.

We parameterize the model for a base case using program evaluation data observed during large- scale pilot CrAg screening programs in Gauteng, Free State, and Western Cape Provinces [19]. Additional parameters related to CD4 testing, patient behavior, and costs of CrAg screening are drawn from existing literature and information from the National Health Laboratory Service (NHLS). For certain parameters, good data do not exist. Therefore, we complete a series of sensitivity analyses to consider implications of varying key parameters, such as CrAg prevalence and adherence to screening guidelines (e.g., patients returning for CD4 results, providers ordering CrAg tests when indicated by guidelines).

## Methods

Reflex and provider-initiated strategies were modeled in two stages: the screening stage and the treatment stage. The screening stage identifies two main categories of patients for the treatment stage: (i) incident CrAg positive patients identified for preemptive treatment; and (ii) incident CrAg positive patients missed by the screening program who also develop CM (discussed in more detail below).

In the treatment stage, costs include outpatient fluconazole treatment for patients identified for preemptive treatment in the screening stage. Treatment costs also include hospitalization and post-hospital treatment for patients who develop CM, which includes some patients missed by the screening program and patients identified for preemptive treatment who may still develop CM. A patient’s outcome in the screening stage determines where they enter the treatment stage. The structure of the treatment cost module is the same for both strategies; only the starting numbers of patients in each arm of the model differ (based on the results of the screening program).

A key starting point for this analysis is CrAg prevalence in the target population at the time of CD4 count testing. While CrAg prevalence in some populations has been reported in many prior studies [6, 7, 9, 20, 21], the distribution of CrAg positive patients not yet on ART with CD4 counts <100 cells/ μl will include incident cases as well as those with prior, treated CM. Among the CrAg positive incident group (the target group for preemptive treatment), some will have low fungal burden at time of screening (and no evidence of cerebrospinal fluid (CSF) disease) and be good candidates for preemptive fluconazole treatment. Others will have a high fungal burden with likely meningitis already at the time of screening and fare poorly on preemptive treatment. For the base case, CrAg prevalence and provider and patient adherence parameters were based on data reported from an evaluation study of CrAg screening programs in South Africa [19, 22].

The remainder of this section works through the screening and treatment cost modules. Tables 1 and 2 and S1 Appendix provide all information not included in screening and treatment flowcharts discussed below. Excel was used for all calculations.

**Table 1:** Distribution of CrAg status in population.

	Prevalence based on test results
Proportion CrAg-	0.954
Proportion prior CM	0.016
Proportion incident CrAg+	0.030
Among incident CrAg+	
proportion CSF+ at time of screening	0.330
proportion CSF- at time of screening	0.670
Among incident CrAg+ but CSF-	
proportion develops CM if not initiated on ART	0.900
proportion develops CM if initiated on ART	0.660

**Table 2:** Basic unit costs and model assumptions.

Year for analysis	2015		
Types of costs	Rand	USD	Notes
Unit cost of CrAg test (reflex)	75.00	4.95	Rand.
Unit cost of CrAg test (provider-initiated)	93.75	6.19	Rand (25% above reflex, as explained in text).
Unit cost (200 mg fluconazole tablet)	0.86	0.06	See S1 Appendix.
Preemptive fluconazole treatment (outpatient)	455.72	30.09	See S1 Appendix.
Hospitalization and treatment for cryptococcal meningitis	29535.00	1,950.10	See S1 Appendix.
Post-hospital maintenance fluconazole treatment (outpatient)	405.84	26.80	See S1 Appendix.
**Other**			
Life expectancy at death (death at age 35–39)	32.6		World Health Organization’s Global Health Observatory
Disability adjusted life years lost per death (3% discount rate)	21.6		Calculated by authors using discrete time approach [24].
2015 exchange rate (ZAR/USD)	15.15		Oanda.com (December 14, 2014–2015)

### Reflex Screening

**Fig 1:**
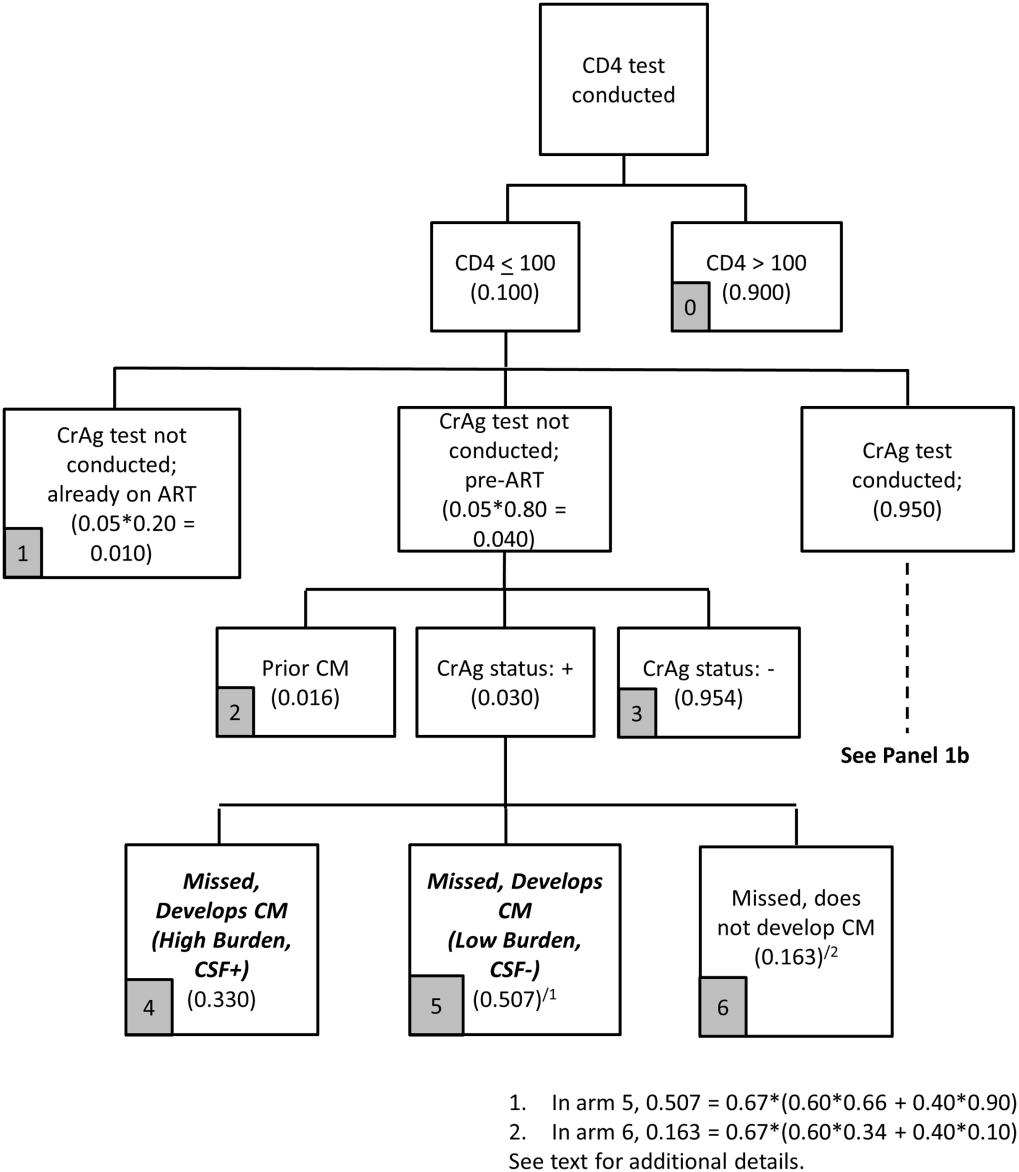
Reflex screening flowchart (Panel 1a).

**Fig 2:**
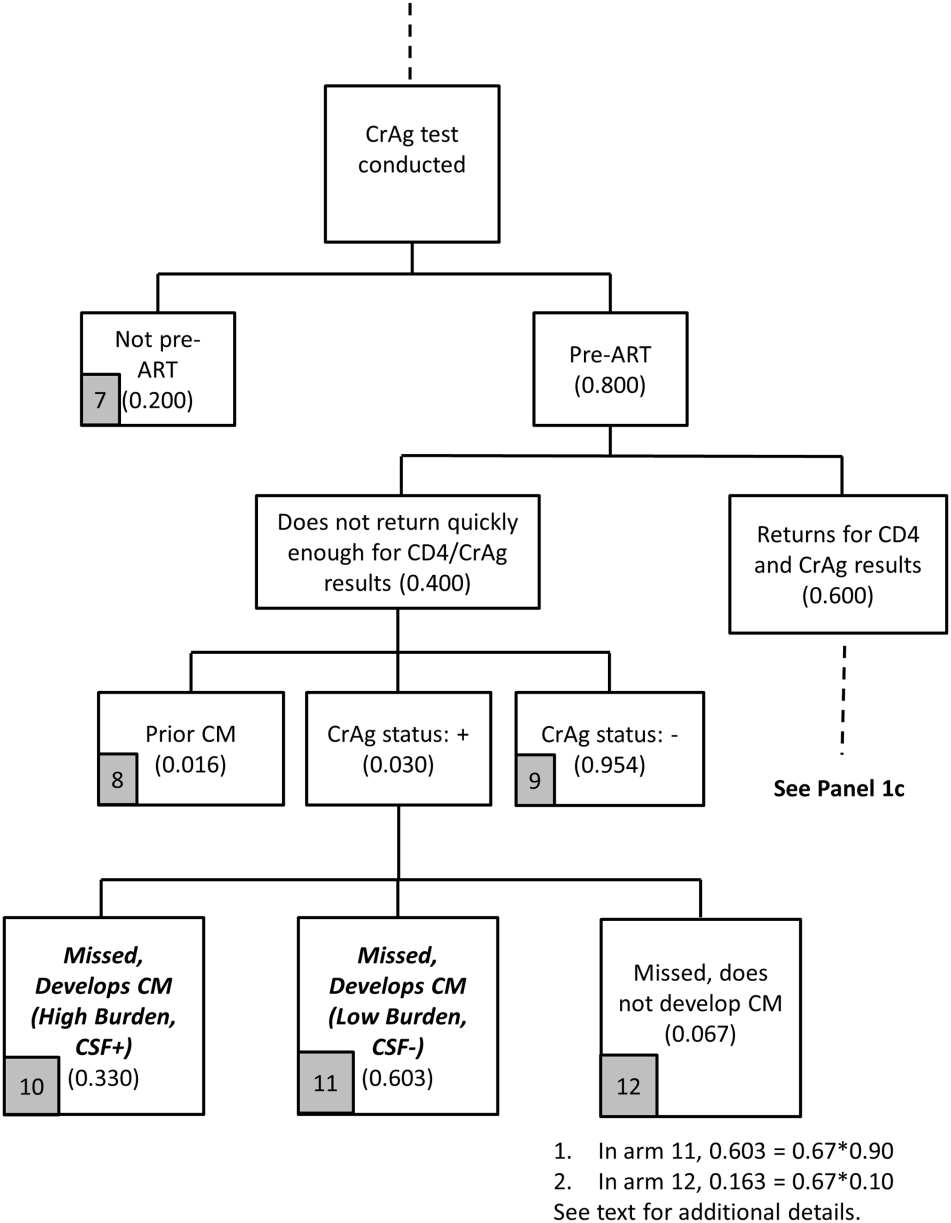
Reflex screening flowchart (Panel 1b).

**Fig 3:**
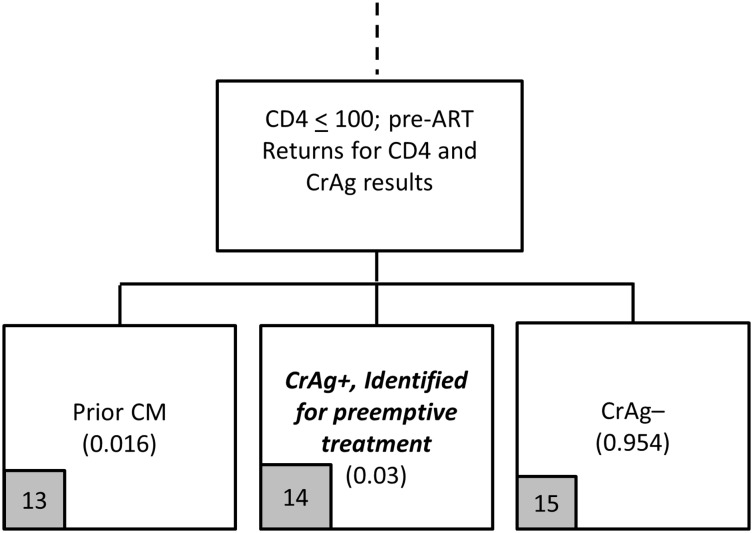
Reflex screening flowchart (Panel 1c).

The screening stage for the reflex strategy is described in Figs 1–3. Parameter assumptions for the base case scenario are included in parentheses in these figures, with additional details provided in Table 1.

Under the reflex strategy, 10% of blood samples have a CD4 count < 100 cells/uL; 95% of these are tested for CrAg, with the remaining 5% not tested due to non-adherence to guidelines by lab personnel (Fig 1). In 2014, the NHLS completed about 3.9 million CD4 count tests, and 9.3% (362,000) specimens had a CD4 count < 100 cells/μl [23].

Among the 5% of samples that are not tested, the base case assumption is that 80% are from patients who are pre-ART (and 20% are from patients already on ART). This is based on CD4 results from eight Right to Care-supported public clinics in Gauteng Province from 2013. Although the South African CrAg screening guidelines specifically target patients who are not yet on ART, reflex lab screening procedures do not identify patients with low CD4 counts who are on ART. Since patients on ART are not the intended targets for CrAg screening (Fig 1, arm 1), they are excluded from further analysis in the model (i.e., they incur screening costs but do not contribute to estimates of health outcomes or treatment costs).

Among those not screened, we use existing program data to estimate that 95.4% are CrAg negative (Fig 1, arm 3), 1.6% are patients with prior CM (arm 2), and 3.0% are incident CrAg positive cases. The screening program misses these incident CrAg positive patients.

Among these CrAg positive patients who are missed by the screening program, because they were not screened, the base case assumption in Table 1 is that 33% have a high fungal burden, are already CSF positive at time of screening, and will develop CM (Fig 1, arm 4). Among the 67% with low fungal burden and CSF negative at time of screening, we assume that 60% initiate ART quickly and 40% do not. Among those who initiate ART when CSF negative, the base assumption is that 66% develop CM in the absence of fungal treatment. Of those who do not initiate ART when CSF negative, 90% will develop CM. Combining the CSF negative patients who do and do not initiate ART, 50.7% of CrAg positive patients missed by the screening program will develop CM (Fig 1, arm 5). The remaining 16.3% of CrAg positive individuals missed do not develop CM (Fig 1, arm 6).

Actual data on the proportion of CrAg positive individuals, with low CD4 counts and CSF negative at the time of CD4 testing, who then develop CM does not exist for a large population of HIV-infected patients (whether there is rapid initiation of ART or not). The implications of these assumptions are explored further through sensitivity analysis following the presentation of main results.

Among the 95% of reflex samples tested for CrAg (Fig 2), 80% are assumed to be from patients who are pre-ART. The question is: do these patients return ‘quickly enough’ to potentially benefit from initiation of preemptive treatment? The definition of ‘quickly enough’ is intended to recognize that CrAg positive patients need to be identified early enough for preemptive treatment to be successful (so CM and hospitalization is avoided). This time period is not well defined in the literature and varies depending on CrAg titer at the time of the CD4 count/CrAg tests (which is unknown with the qualitative CrAg test in use in South Africa). South African CrAg program evaluation data suggest that, of the 80% of patients who returned for CD4 results after HIV testing, 75% returned within 28 days. Thus, our base assumption is that 60% (75%*80%) return quickly enough, defined as ≤ 28 days, and 40% do not. These numbers are also consistent with prior literature evaluating losses to follow up after initiating CD4 testing [14, 18].

For the base case (Fig 2), among the 40% of patients who do not return quickly enough, 3% are CrAg positive and missed by the screening program. Because they do not return for ‘quickly enough’ for CD4 results, we therefore assume they do not initiate ART quickly enough for ART-induced immune recovery alone to reduce their risk of progression to CM. As in Panel 1a, we assumed that 33% are CSF positive at the time of screening and 67% are CSF negative. We assume that 90% of missed patients who are CSF negative will develop CM (so 0.90*0.67 = 60.3% of all CrAg positive patients who did not return for their CD4 result will develop CM).

Among the 60% of pre-ART patients who return ‘quickly enough’ to the clinic for a second visit, 95.4% are CrAg negative, 1.6% are positive and have had prior CM, and 3% are incident CrAg positive patients (Fig 3). The incident CrAg positive patients (Fig 3, arm 14) are the intended targets of the screening program actually identified by the screening program; those with prior CM (Fig 3, arm 13) happen to be screened due to the reflex policy and incur screening costs (but have no further health outcomes or treatment costs in this analysis).

Based on the model outlined in Figs 1–3, the reflex screening program incurs screening costs and identifies two main categories of patients for the treatment cost module: (i) patients missed by the screening program who develop CM (arms 4, 5, 10, 11); and (ii) patients identified for preemptive treatment (arm 14). The cost of screening is simply the unit cost of a CrAg test, with a base case of $4.95 per test (year of analysis is 2015, see Table 2).

### Provider-initiated Screening

**Fig 4:**
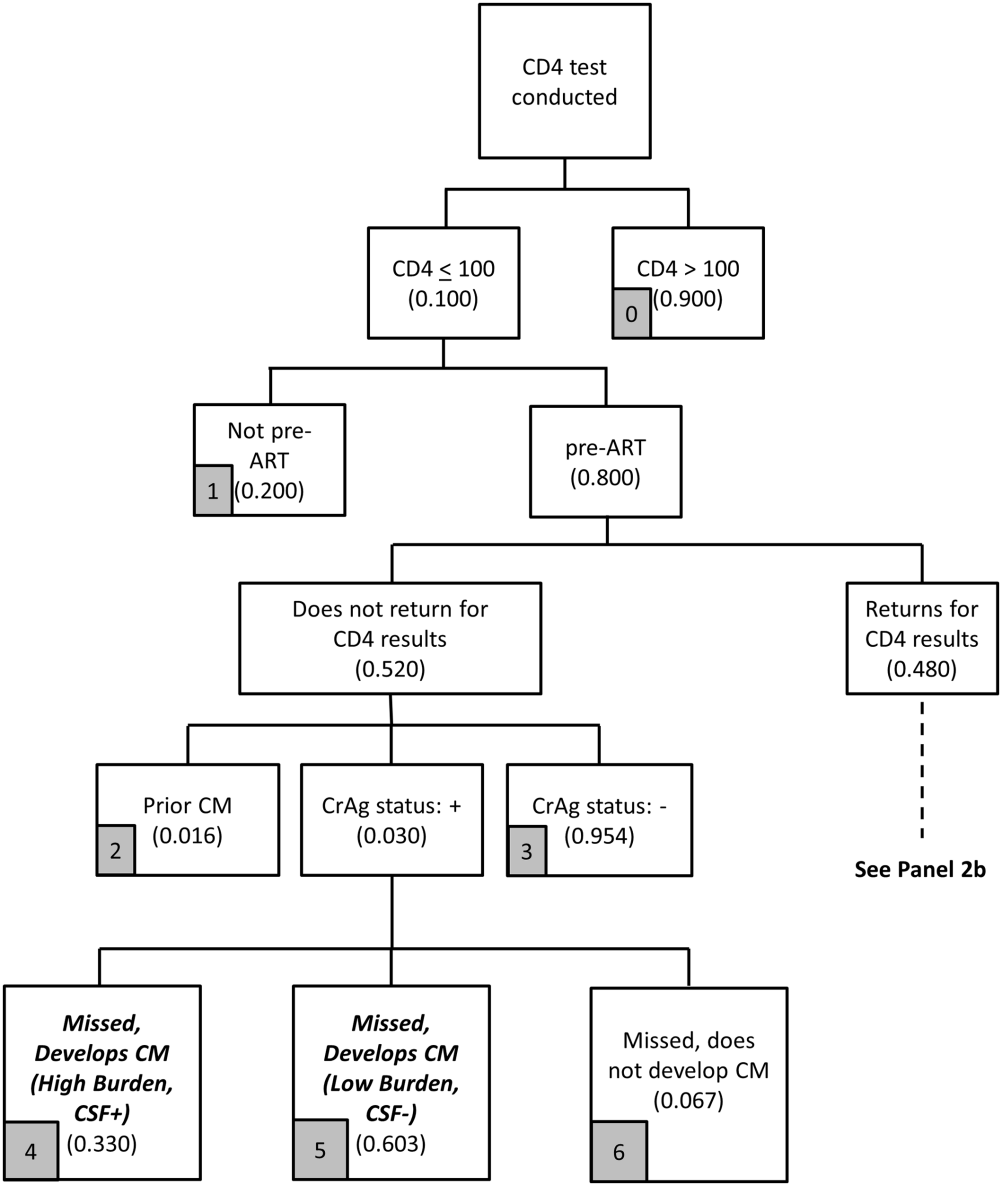
Provider-initiated screening flowchart (Panel 2a).

**Fig 5:**
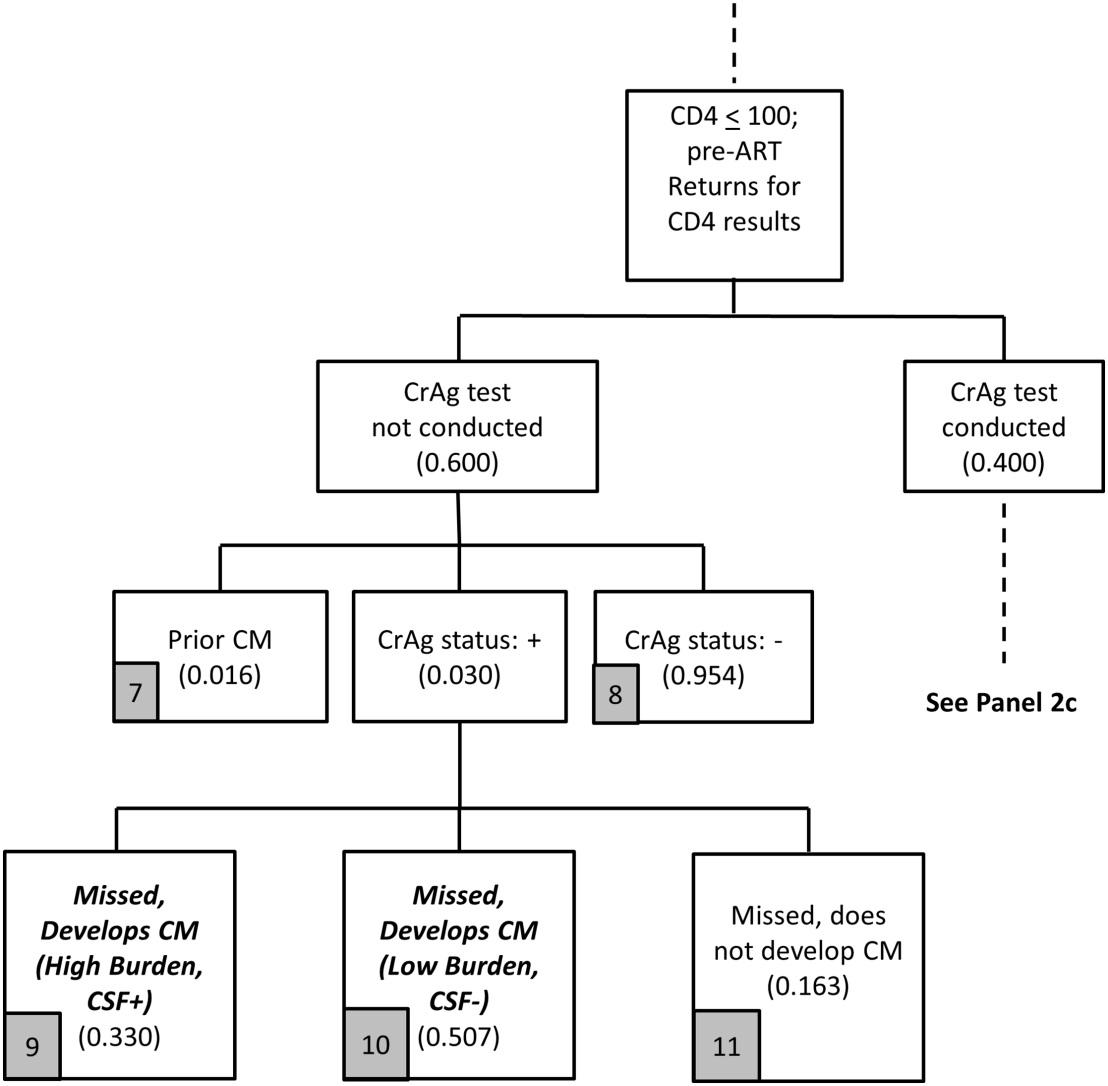
Provider-initiated screening flowchart (Panel 2b).

**Fig 6:**
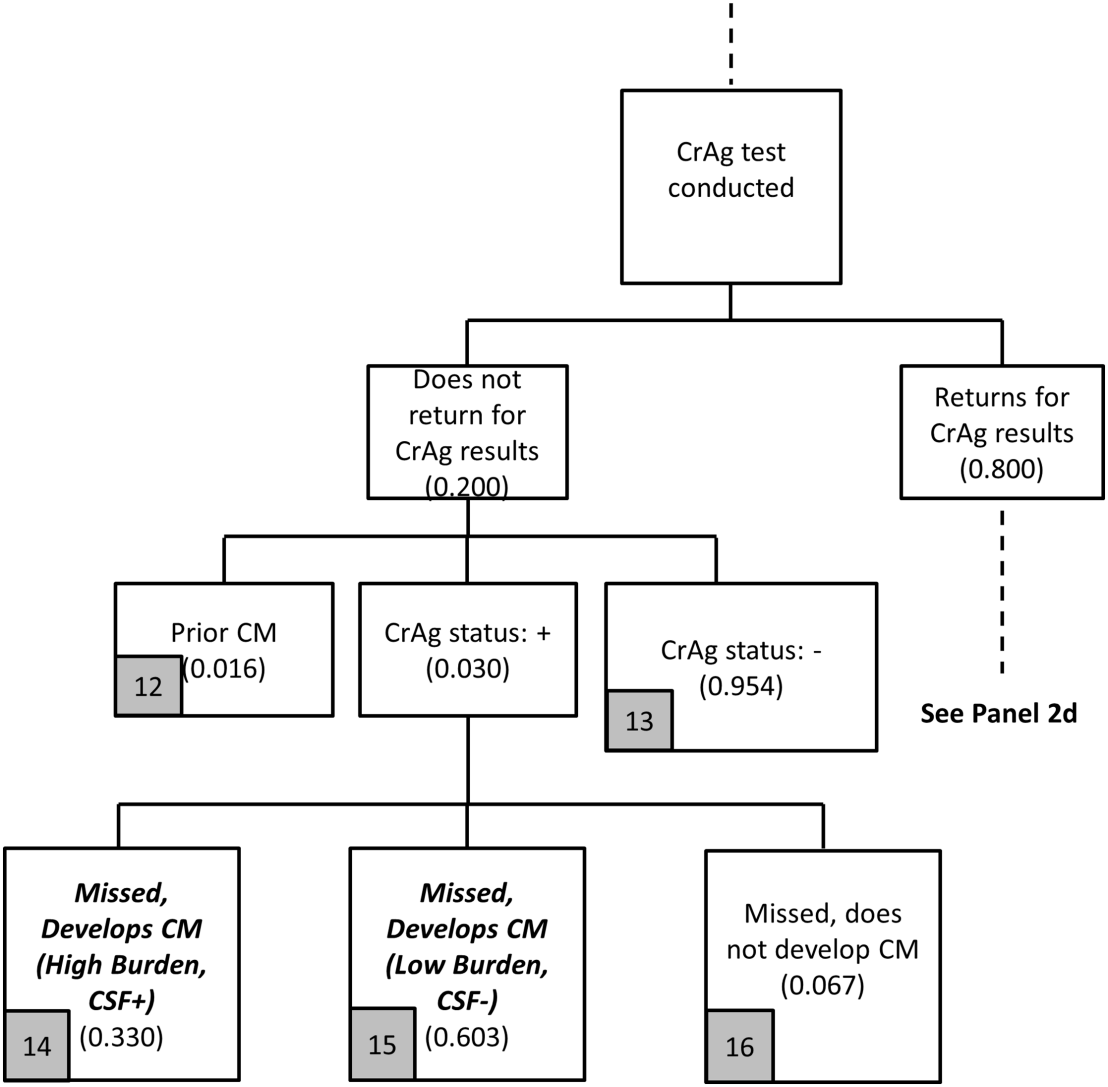
Provider-initiated screening flowchart (Panel 2c).

**Fig 7:**
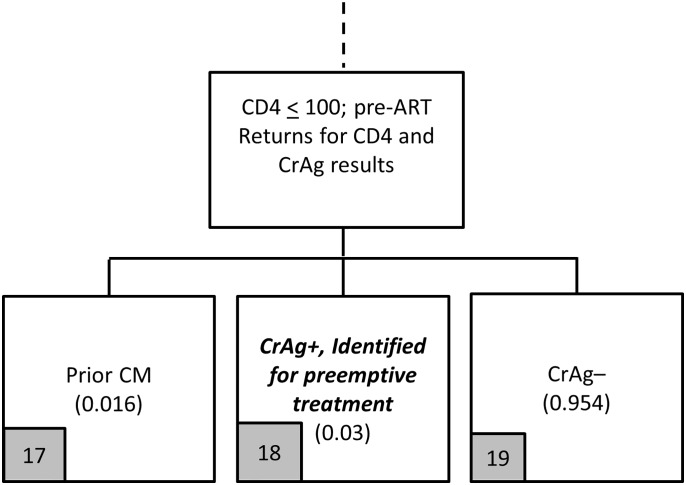
Provider-initiated screening flowchart (Panel 2d).

The logic of the provider-initiated screening module, presented in Figs 4–7, is similar to the reflex screening analysis but with a few key adjustments due to the structure of provider-initiated screening.

The first key difference between the two strategies is that, with provider-initiated screening, patients need to return ‘quickly enough’ for their CD4 results, and also then return ‘quickly enough’ to collect CrAg results (two return visits to complete CrAg screening are needed with the provider-initiated policy rather than the one needed for the reflex policy). In Fig 4, as with the reflex policy, 10% of blood samples sent for CD4 testing have a CD4 count < 100 cells/μl, and 80% of these patients are not yet on ART. In Fig 4, the base case assumes that 48% of patients return quickly enough for CD4 results, so that they could return again quickly enough for CrAg results, with both visits completed within a total of 28 days (to be consistent with the reflex policy). For reference, 48% is consistent with the assumption that 60% of patients return within 28 days (as with the reflex policy), and 80% of these patients return quickly enough (60%*80% = 48%) to be able to collect CrAg test results within 28 days.

A second key difference between the two screening strategies is that provider-initiated screening allows clinicians to target CrAg screening to the intended group of patients: those who are pre-ART and with CD4 count < 100 cells/μl. In Fig 5, only these patients are screened; there are no costs associated with screening patients already on ART. In theory, providers would discuss CrAg screening with patients, and any patient with a known history of prior CM would not be screened for CrAg. However, based on existing program experience, providers may still request the test and, therefore, some of those screened for CrAg will be patients with prior CM.

A third key difference between the two strategies is provider adherence to screening guidelines. With the reflex policy, 95% of all patients who should be screened for CrAg are screened (in addition to patients who are not the target of the screening program), and CD4 count and CrAg results are available to the clinician simultaneously. With the provider-initiated policy, which depends on provider decision-making, such high levels of adherence to guidelines cannot be assumed. Experience from the Western Cape Province, which implemented the provider-initiated policy, showed low adherence to guidelines, with some modest improvement over time (< 35% across all time periods) [25]. Assuming that some improvement could be obtained over time with better training, the base case assumption is that providers request the CrAg test on 40% of patients who should receive the test; that is, 40% of the pre-ART patients with CD4 count < 100 cells/μl who return quickly enough to learn their CD4 results receive CrAg testing by providers.

Returning to Fig 4, among the 52% of patients who do not return to the clinic to learn their CD4 test results, we again assume that 3.0% are incident CrAg positive, 1.6% have had prior CM, and 95.4% are CrAg negative. As in Figs 1–3 for reflex screening, 33% of the incident CrAg positives patients will already be CSF positive and develop CM (Fig 4, arm 4). Among the 67% who are CSF negative, since these patients are also unlikely to initiate ART quickly because they did not return for CD4 results, 90% of these patients are assumed to develop CM.

Of the 48% of patients with CD4 count < 100 cells/μl who return for CD4 results, providers request CrAg tests on 40% of these patients. For the remaining 60% not tested for CrAg, again 3% are incident CrAg positive (Fig 5). As with reflex screening, 33% of the incident CrAg positives patients will also already be CSF positive and develop CM. Among the 67% who are CSF negative, since these patients returned for CD4 results (but then did not receive a CrAg test), some patients initiate ART and some do not, so the proportions in Fig 5 (arms 9–11) are identical to the proportions in Fig 1 (arms 4–6).

The patients actually screened for CrAg need to return quickly enough (their third visit to the clinic within 28 days) to obtain their CrAg results. Data are currently not available to be able to provide a reasonable estimate for this parameter. As a base case, we assume that 80% of these patients return within 28 days, and 20% do not. A high proportion, such as 80%, seems plausible because these are patients who already returned quickly for CD4 results, know their low CD4 results, and are likely to be motivated to return again quickly.

Among patients tested for CrAg who do not return quickly enough (20% of those tested), 3.0% are incident CrAg positive with the same structure of outcomes (missed and developed CM) as in Fig 6 as in Fig 4. For the 80% of patients who do return quickly enough for their CrAg results with provider-initiated screening (Fig 7), they have the same overall experience as those under the reflex strategy: 3.0% are incident CrAg positive, 95.4% are CrAg negative, and 1.6% have had prior CM.

Based on the model outlined in Figs 4–7, the provider-initiated strategy identifies two main categories of patients for the treatment cost module: (i) patients missed by the screening program who develop CM (arms 4, 5, 9, 10, 14, 15); and (ii) patients identified for preemptive treatment (arm 18). The cost of screening is again simply the unit cost of a CrAg test completed as a stand-alone test, which is discussed in more detail in the section below on unit costs.

Far fewer patients will be screened for CrAg with the provider-initiated policy in part because screening is avoided for patients already on ART. In addition, patients who should be screened are not because they do not return for CD4 results and providers do not order the CrAg test.

### Treatment

**Fig 8:**
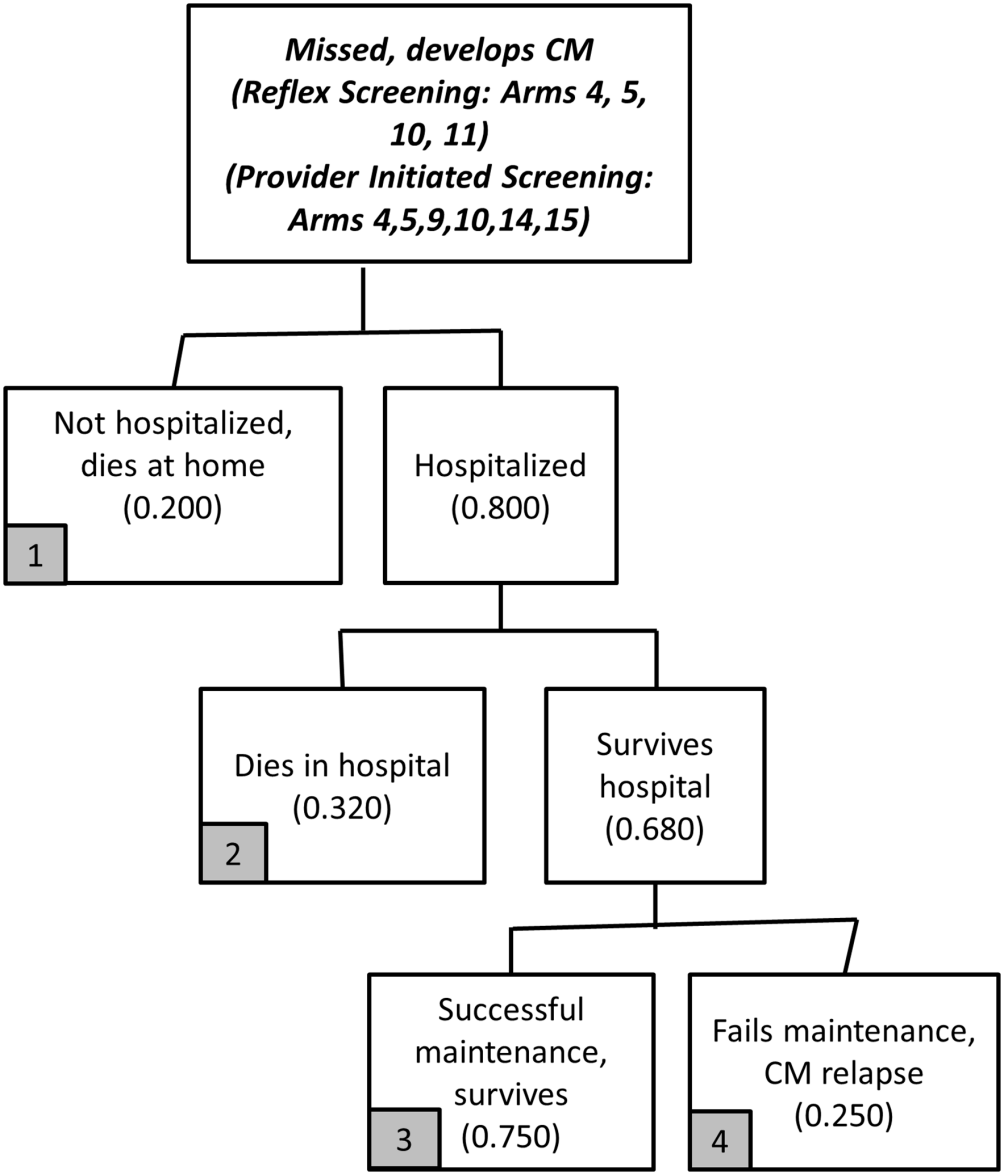
Treatment model flowchart (Panel 3a).

**Fig 9:**
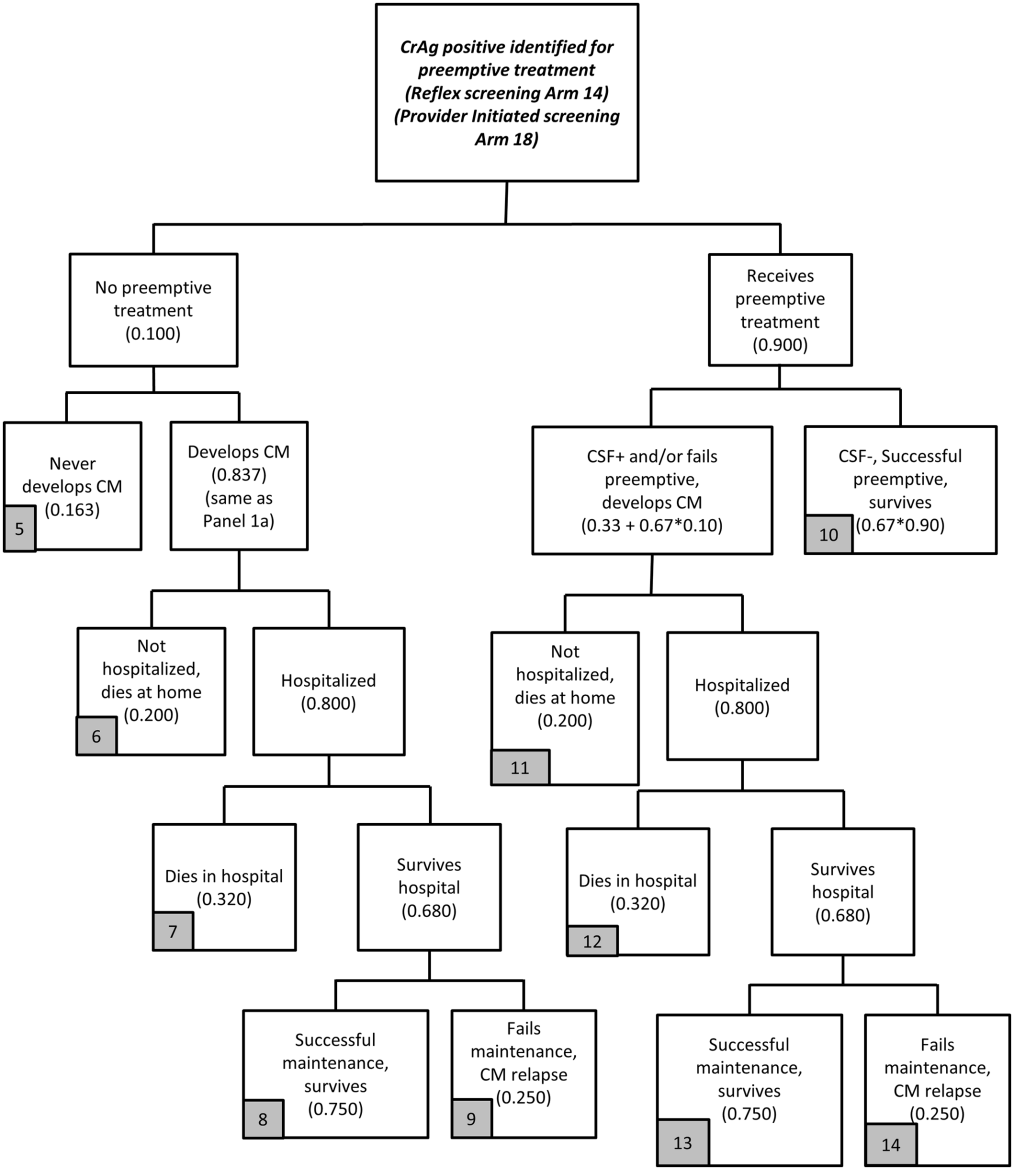
Treatment model flowchart (Panel 3b).

The treatment stage is presented in Figs 8 and 9. Additional basic information on treatment costs is provided in Table 2 (and S1 Appendix). The treatment module is organized into two main treatment arms, one for each of the categories of patients identified in the screening modules: (i) CrAg positive patients *missed* in the screening stage who develop CM (Fig 8); and (ii) CrAg positive patients identified for preemptive fluconazole treatment (Fig 9). The structure of the treatment cost module is the same regardless of screening strategy, but the starting numbers of patients in each category (incurring treatment costs and health outcomes) are derived from the screening strategy.

Care for CrAg positive patients missed during screening who develop CM is described in Fig 8. Among these patients who develop CM, 80% are hospitalized with CM, and 68% survive hospitalization. Among those who survive hospitalization, 75% will be successful on maintenance fluconazole and 25% will fail maintenance treatment and have CM relapse. The 20% with CM not hospitalized die at home.

The treatment cost module for CrAg positive patients identified for preemptive treatment is described in Fig 9. While preemptive treatment with fluconazole is indicated for all of these patients, 10% do not receive preemptive treatment, whether due to failure to initiate fluconazole by providers, pharmacy stock-outs, or patient behavior.

Among those that do not receive preemptive treatment, most will develop CM. Using the same logic as in Fig 1 (arms 4–6), the base case assumption is that 16.3% will never develop CM while 83.7% develop CM (accounting for those already CSF positive and those who are CSF negative but develop CM regardless of ART status). Of the 83.7% who develop CM, we assume that 80% will eventually be hospitalized for CM, with 68% surviving hospitalization and 32% dying in the hospital [26]. The 20% with CM not hospitalized also die. Among those who survive hospitalization, 75% are assumed to be successful on post-hospitalization maintenance fluconazole treatment, while the remaining 25% will fail maintenance treatment and have a relapse of CM. Although good data do not exist on some of these parameters, because the focus here is on the comparison of the two models, changes in these assumptions (e.g., 80% hospitalized to 50% or 100%) will affect treatment costs and outcomes for both strategies in the same way (hospital costs increase or decrease for both strategies, lives saved increase or decrease).

For this analysis, a relapse of CM is an endpoint, and we do not include additional treatment costs for these patients or include them in the outcome estimates (e.g. lives saved). A small number of patients have this endpoint, so the additional treatment costs and health outcomes are essentially irrelevant for a policy comparison.

Most patients identified for preemptive treatment initiate treatment (90%). Current guidelines in South Africa for preemptive care of identified CrAg positive patients are 800mg of fluconazole for 2 weeks prior to the initiation of ART, followed by initiation of ART and continuation of daily fluconazole, 400mg for 8 weeks and then 200mg daily for at least 1 year until CD4 counts are > 200 cells/μl.[13, 27] The details vary slightly depending on if the patient has symptoms relevant to CM and if the patient receives a lumbar puncture for further diagnosis.

Some incident CrAg positive patients will also receive a lumbar puncture to assess CSF status, whether symptomatic or asymptomatic, after receiving their CrAg test results, at the same visit or a future visit perhaps to another facility.[28] The costs of such lumbar punctures are excluded from the treatment model in large part because the additional cost of a lumbar puncture is small (see, e.g., [12]) and the result is basically the same (they are CSF positive at the time of initiating preemptive treatment).

Among the patients initiated on preemptive fluconazole, some will be treated successfully and some will fail on preemptive treatment and develop CM (or be diagnosed as CSF positive after a lumbar puncture). Among those initiated on preemptive treatment, an estimated 39.7% will fail preemptive treatment, which combines the 33% of patients already CSF positive at time of preemptive treatment initiation as well as a small share (10%) of those CSF negative. The remaining 60.3% initiated on preemptive treatment will not develop CM. Of the patients who develop CM after initiating preemptive treatment, 80% are hospitalized, of whom 68% survive. Of the patients who survive hospitalization, 75% are successful on maintenance fluconazole. The 20% of patients who fail preemptive treatment, but are never hospitalized and die at home, may have failed on preemptive fluconazole due to being CSF positive at time of fluconazole initiation of lack of patient adherence to fluconazole.

### Costs and health outcomes

Parameter assumptions related to the costs of screening and treatment as well as health outcomes are presented in Table 2 (and S1 Appendix). Based on existing analysis by the NHLS, $4.95 is a best estimate of the average cost of a reflex CrAg test after CD4 testing (across labs of varying daily sample volumes).

Under the provider-initiated strategy, the CrAg test is completed as a separate test, which requires additional provider time, an additional blood draw, additional transport to lab, additional data entry into electronic data systems, etc. Thus, the unit cost for one CrAg test will be higher with the provider-initiated policy. As a base case, we assume a 25% cost differential between the two strategies for a cost of $6.19 for provider initiated CrAg testing.

Patients started on preemptive fluconazole treatment, and who fully adhere to a one-year treatment course, require 533 tablets (200mg each). The cost per 200mg tablet is $0.056, for a total cost of $30.09. The price per tablet is based on the most recent negotiated fluconazole tender in South Africa at the time of this analysis (2015). The cost in USD looks especially low because of the very weak exchange rate in 2015 (ZAR/$15). Not all patients will complete the full year of preemptive treatment. For the base case, partial adherence is defined as taking 66% of the total prescribed annual amount (so 355 200mg tablets at a total cost of $19.86).

For patients with CM successfully treated in the hospital (with 14 days of amphotericin B and related services and hotel costs), we use recently published data on hospitalization costs per day in South Africa to estimate the costs of a full 15-day hospital stay at $1950 ($130 per day). Compared to basic hotel costs, the costs of services provided in the hospital (amphotericin B, lumbar punctures, intravenous saline, etc.) are relatively minor. As an alternative, hospital costs would be slightly lower if we used the detailed information on hospital services and costs reported in Jarvis et al. (see [12]), and then inflated their 2010 values up to 2015 (see S1 Appendix for details).

Those who do not survive the hospital are assumed to require six hospitalized days on average [28], at a total cost of $780. Finally, most patients who survive hospitalization for CM will be started on maintenance fluconazole treatment. Those that fully adhere throughout the one-year treatment course receive 477200mg tablets, for a total cost of $26.9. Partial adherence for maintenance fluconazole is defined as 75% of full adherence, so these patients receive 358200 mg tablets at a total cost of $20.2.

Patients can have three primary outcomes at the end of the treatment module: survived, died, unknown. In the treatment cost module, patients in Fig 8 (arm 4) and Fig 9 (arms 9 and 14) are classified as unknown. Some will die without returning to a hospital. Some of these patients may live long enough to return to a hospital for additional care (and perhaps not die). While additional arms could be developed to allow for such details, the extra complexity provides little additional information for comparing the two policies (overall total costs would increase by a minor amount and deaths would decrease by a minor amount).

For comparing the two screening strategies, health outcomes are based on known lives saved from screening and treatment as well as patients missed by the screening program who do not develop CM. When comparing the two strategies, the difference in this outcome is the additional lives saved from reflex compared to provider-initiated screening. For patients who die from CM, the base case assumption is that the average age of death is between 35–39 years. For this age range, average life expectancy is 32.6 additional years (data from the World Health Organization’s Global Health Observatory). With a 3% discount rate and 32.6 years of life lost from a death, 21.6 discounted years of life are lost per death, or conversely 21.6 years of life are saved per avoided death.

Patients with CM experience serious symptoms and disability whether receiving treatment or not. Thus, in general, the period of time lived disabled due to CM disease prior to and during treatment could be added into the analysis (with disability adjusted life years (DALYs) lost as the combined health outcome). However, the time period for disability for these patients is fairly short (they either respond to treatment or die within one year), so the “years lived disabled” component of DALYs before completion of treatment will be minor for CM compared to the “years of life lost” component (this disability component would add less than 1 to the 21.6 years of life lost already estimated).

In addition, patients who survive their hospital stay and complete the post-hospital fluconazole treatment could have long-term impairment from CM despite surviving. For example, disability weights for meningitis-related conditions used previously in the Global Burden of Disease estimates are, for example, deafness = 0.229; intellectual impairment = 0.456; and motor deficit = 0.380.[29] Such long-term impacts will reduce the estimated health benefits from successful CM treatment to some degree, but are excluded for the base case analysis. The implications of including such long-term disability into the analysis are discussed as part of the sensitivity analysis section following the presentation of the base-care results.

## Results

### Base case model assumptions

In the base case population of 100,000 patients who receive a CD4 test, 8,000 have a CD4 count <100 cells/μl and are not yet on ART. The goal of screening is to identify the 240 patients out of 8,000 who are not yet on ART, with incident CrAg (no prior CM). In the absence of a screening program, all of these patients are missed. In the presence of a screening program, with the base case assumptions and analysis presented in Figs 1–9 and Tables 1, 2 and 3 summarizes screening results (categories of patients identified by the screening strategies) and Table 4 summarizes results related to costs of screening and treatment and health outcomes achieved.

**Table 3:** Outcomes from screening modules for 100,000 patients screened for cryptococcal antigenemia (CrAg).

	RP	PIP
Total Screened	9500	1536
Identified for preemptive treatment	136.8	36.9
Missed and develops CM	95.1	189.5
Missed but does not develop CM	8.1	13.6
Total incident CrAg+	240.0	240.0

**Table 4:** Summary results for base case analysis (per 100,000 CD4 tests).

	Reflexive Policy	Provider-Initiated Policy	Difference (RP—PIP)
Number screened	9,500	1,536	7,964
Total cost of screening (2015 USD)	$47,044	$9,508	$37,536
Total cost of treatment (2015 USD)	$209,399	$264,564	-$55,165
Total cost of screening + treatment (2015 USD)	$256,443	$274,072	-$17,629
Total number of patients known to survive (from treatment model plus those who survive but are missed and do not enter treatment model)	148	118	30
Total years of life saved from surviving patients	3,189	2,542	647
Cost per additional life year saved (RP compared to PI, USD)			-$27.25

### Costs and outcomes of screening

From Table 3, the reflex policy correctly identifies substantially more patients who are the target of the screening program than the provider-initiated approach (137 compared to 37 out of the 240 patients, respectively). An important share of patients missed by the screening program, as well as those identified for preemptive treatment, are already CSF positive, although this information is not known at the time of screening. We consider these patients missed by the screening program, but perhaps a better interpretation is that these patients presented for HIV care too late in their disease progression to benefit from preemptive treatment.

From Table 4, 9,500 patients are screened with the reflex policy for a total cost of $47,044. With the provider-initiated policy, 1,536 patients are screened for a cost of $9,508. While the reflex policy screens more patients that are not the target of the policy, the provider-initiated policy misses a large share of the target patients because a large share of patients do not return for CD4 results, and then providers fail to order CrAg testing for a large share who do return.

### Costs and outcomes of treatment

In the base case summarized in Table 4, reflex screening dominates provider-initiated screening. The cost of screening is substantially higher with the reflex policy compared to the provider-initiated policy ($37,536 more), but the cost of treatment (preemptive, hospitalization, and post-hospital maintenance) is substantially lower ($55,165 less), so that the total cost of screening and treatment per 100,000 CD4 tests for the reflex strategy is $17,629 less than for the provider-initiated strategy.

In terms of health outcomes, the reflex policy is also estimated to save an additional 30 lives per 100,000 CD4 tests (647 additional years of life saved). Note that these are estimates of additional patients “known” to be living at the end of the treatment stage; any patients in the treatment arms with indeterminate final health outcomes (CM relapse) are not included in this estimate, but their treatment costs are included. For reference, there are 21 patients in the reflex policy treatment module and 28 patients in the provider-initiated treatment module with this indeterminate health outcome (Fig 8, arm 4 and Fig 9, arms 9 and 14).

Table 5 provides additional information on the cost of treatment, disaggregated into preemptive fluconazole treatment, hospitalization, and post-hospital maintenance treatment. For both policies, the majority of treatment costs are associated with hospital-based treatment for CM. The reflex policy is able to identify more patients for preemptive treatment in the base case, so that cases of CM are avoided. However, a substantial number of patients are missed and develop CM with both policies as described above, so that hospitalizations are not avoided with either policy.

**Table 5:** Disaggregated costs (2015 USD) and quantity of fluconazole tablets (200 mg) per 1000,000 CD4 tests.

	Reflex Policy (RP)	Provider-Initiated Policy (PIP	Diff (RP-PIP)
Tablets of fluconazole for preemptive treatment	40,967.94	11,039.78	29,928
Tablets of fluconazole for maintenance treatment	38,893.65	50,086.39	-11,193
Tablets of fluconazole total	79,861.59	61,126.17	18,735
Cost of preemptive fluconazole treatment	2,312.75	623.23	1,690
Cost of in-patient hospitalization	204,890.77	261,113.30	-56,223
Cost of maintenance fluconazole treatment	2,195.65	2,827.52	-632
In-patient costs as share of total costs (treatment + screening)	0.80	0.95	

### Sensitivity analyses

The screening and treatment model can be easily used for sensitivity analyses or for applications in other settings (all unit costs would need adjustment for other countries). We first consider 5 initial scenarios (denoted as SA1-SA5) beyond the base case, and key results are summarized in Table 6. SA1 addresses better provider adherence with requesting the test when indicated in provider-initiated screening (from 40% to 80%), which only affects provider-initiated results; SA2 includes better patient adherence with returning for CD4 results (from 60% to 80% for reflex screening; and 52% to 64% for provider initiated). Reflex screening continues to dominate provider-initiated screening for both scenarios. The same results hold with higher incident CrAg prevalence (e.g., 3% to 6%). In addition, even if a large proportion of surviving CM patients has long-term impairment, the reflex screening generates more health outcomes compared to the provider-initiated screening.

**Table 6:** Sensitivity analyses (all cost in 2015 USD, per 100,000 CD4 tests)^a^.

	Base case	SA1	SA2	SA3	SA4	SA5
Percentage screened as CrAg positive	0.03					
Provider adherence: Percentage of indicated tests requested with provider-initiated screening	0.40	0.80	0.80		0.80	0.80
Patient adherence: Percentage of patients return for CD4 results	0.60		0.80			0.80
Proportion CSF negative that develops CM if not initiated on ART	0.90			0.45	0.45	0.45
Proportion of CSF negative that develops CM if initiated on ART	0.66			0.33	0.33	0.33
Outcomes: Reflex compared to provider-initiated strategy (only compare numbers in the same column)						
Additional number of patients screened	7,964	6,428	5,404	7,964	6,428	5,404
Additional cost of screening (2015 USD)	37,536	28,028	21,690	37,536	28,028	21,690
Additional cost of treatment (2015 USD)	-55,165	-35,358	-46,654	20,960	12,957	17,521
Additional cost of screening + treatment (2015 USD)	-17,629	-7,330	-24,964	58,496	40,986	39,211
Additional patients known to survive	30	19	25	24	15	20
Additional years of life saved	647	414	547	522	332	441
Additional cost (2015 USD) per additional year of life saved (reflex compared to provider initiated)	-27	-18	-46	112	123	89

^a^ Empty cell means the number is identical to the base case assumption.

As noted in Table 6, the base case model assumes that 90% of incident CrAg positive patients who are CSF negative at the time of screening develop CM if not initiated on ART quickly (and 66% will develop CM if initiated on ART). In Table 6, scenarios SA3-5 show how results change if substantially fewer incident CrAg positive (but CSF negative) individuals progress to CM (a 50% reduction in both parameters). If a large proportion of these individuals never develop CM, reflex screening compared to provider-initiated screening incurs more screening costs but does not save on hospital costs because a large proportion of patients missed by provider-initiated screening never develop CM. As a result, reflex screening does not dominate provider-initiated screening, but the cost per additional life year saved is modest (<$125). If, in addition, a large proportion of surviving CM patients has long-term impairment, the cost per additional life year saved would increase (e.g., <$250 with a large proportion patients with serious chronic impairment).

## Discussion and Conclusion

Compared to no screening, previous studies have shown that CrAg screening prior to the initiation of ART for patients with baseline CD4 counts < 100 cells/μl is a relatively low cost or cost-saving policy that also saves lives (11,12). This will remain true as long as a baseline CD4 count continues to be required prior to the initiation of ART, even if in the future, CD4 thresholds disappear as an eligibility criterion for ART initiation.

The question addressed in this analysis is: how best to organize a screening program in a given setting? The model developed for this analysis and the results reported in Tables 4 and 5, along with additional sensitivity analyses reported in Table 6, suggest that reflex screening compared to provider-initiated screening is a relatively low cost or perhaps cost saving policy that also saves lives.

As with any policy, efficient operational implementation is critical to achieving program targets. In particular, the important first step is to support patients to know their HIV status and present for care when their CD4 count is still well above 100 cells/μL. The second key step is to get patients to return for CD4 results relatively quickly. In South Africa, patients are typically counseled to return in one week, which if adhered to, provides a good opportunity for CrAg-positive patients to benefit from early treatment. Given that NHLS typically completes CD4 tests within 48 hours, opportunities to shorten the return-for-CD4 results period could be evaluated further.

Third, screening and provision of preemptive fluconazole treatment must be simultaneously implemented. While a relatively small number of NHLS laboratories provide the bulk of CD4 tests completed in the country, thousands of Primary Health Centers (PHCs) initiate patients on ART. Based on estimates of 200 mg fluconazole tablets needed per 100,000 CD4 tests in Table 5, with 3.9 million CD4 count tests completed in NHLS laboratories in 2014, approximately 3.2 million tablets need to be procured and managed through supply chains to ensure adequate stocks over time in these thousands of PHCs.

As with most modeling analyses, limitations follow from the need to develop model parameters based on data from several sources (and rough estimates when data are especially lacking). As much as possible, parameters for this analysis were generated from a recent evaluation of CrAg screening programs in South Africa [19]. As shown with the sensitivity analyses in Table 6, however, changing key parameters related to CrAg prevalence, patients returning for CD4 results, and providers requesting CrAg tests, does affect the costs and magnitude of outcomes achieved with both screening strategies. Reflex screening generally dominates provider-initiated screening or has a low incremental cost-effectiveness ratio as long as a large share of incident CrAg positive patients develops CM in the absence of anti-fungal treatment. When a small share of incident CrAg positive patients develop CM in the absence of anti-fungal treatment (see Table 6, SA3-5), reflex screening costs more overall (screening plus treatment) but continues to have a low incremental cost-effectiveness ratio (< $125 per life year saved).

Besides the reflex and provider-initiated CrAg screening policies currently included in South Africa’s HIV guidelines, additional strategies have been discussed or may be relevant in the future. First, for example, point-of-care (POC) CrAg screening as part of a provider-initiated approach, using a lateral flow assay (LFA) method when a patient returns for CD4 results, is possible. However, unless paired with a POC CD4 test, this approach does not provide CrAg results to patients any sooner than the reflex policy, but would eliminate the need for a third return visit under a provider-initiated policy. While the CrAg LFA can be performed relatively easily as a POC test, a large scale program of training, quality control, supply chain management, and data systems would need to be developed for POC CrAg screening to be implemented across thousands of PHCs with equivalent quality to current lab-based CrAg screening [30]. The additional costs from reflex lab screening of patients already on ART and/or with a history of CM are likely to be relatively minor compared to the costs of developing a national POC CrAg screening program.

Second, in the future quantitative CrAg tests, reporting a CrAg titer to better differentiate disease status, may help target those who should receive preemptive treatment with fluconazole and who should be admitted and treated with amphotericin. While beyond the scope of the current paper, the existing model could be adapted to such strategies for assessing costs and effectiveness.

And third, all patients testing positive for HIV could be prescribed an initial short-course of fluconazole, or perhaps only patients clinically suspected to have a low CD4 cell count based on WHO staging or some other criteria, while awaiting CD4 results. When these patients return for their CD4 result and CrAg result (if CD4 < 100 cells/μl and with reflex screening), those testing CrAg negative would stop fluconazole treatment and immediately initiate ART, while those CrAg positive would continue with preemptive treatment prior to initiating ART. Future research is needed to evaluate the health risks and benefits of such an approach. However, the logic of this third approach becomes more relevant if CD4 test results are not required *prior* to the initiation of ART as is the case for countries considering “test and treat” policies.

CrAg screening prior to the initiation of ART is a relatively low cost or potentially cost saving policy *as long as* a baseline CD4 count continues to be included in national guidelines prior to the initiation of ART. Even if all HIV patients are eligible for ART, regardless of CD4 counts, the question remains if a baseline CD4 count will be required prior to initiation of ART or not.

If guidelines under new test-and-treat policies still include baseline CD4 counts and other laboratory tests *prior to initiation* of ART, then a reflex CrAg screening strategy imposes no additional delays on ART initiation for the vast majority of patients. For the relatively few total HIV-infected patients with low CD4 counts who are CrAg positive, appropriate treatment (preemptive fluconazole or hospitalization) can still begin prior to initiation of ART.

However, CrAg screening *prior to* the initiation of ART becomes more complicated if policies change to same-day ART initiation after an HIV diagnosis (or at initial presentation to a PHC if tested in a mobile testing program). If an immediate test-and-treat approach is implemented as a national policy, CrAg screening *before initiation* of ART would require both provider-initiated POC CD4 and CrAg testing. Alternatively, CrAg screening *after initiation* of ART (perhaps implemented discussed above) would need to be evaluated further.

To date, all analyses of CrAg screening have assumed the presence of CD4 results to target screening. In the absence of such information, future research is needed to compare non-CD4 based strategies for screening, as well as the benefits of CrAg screening for the small proportion of newly-diagnosed HIV-infected patients with low CD4 counts who are incident CrAg positive to the possible costs of delaying ART initiation for the vast majority of all patients (those with CD4 counts > 100 cells/μl and those with low CD4 counts who are CrAg negative).

### Ethics statement

This costing analysis was based on routinely-collected aggregated program data, publically available information, and aggregated results from evaluation studies. No human subjects data were used.

## Supporting Information

S1 Appendix Treatment cost calculations in detail. (DOCX)
