# Supplementary material for: Screening HIV-Infected Patients with Low CD4 Counts for Cryptococcal Antigenemia prior to Initiation of Antiretroviral Therapy: Cost Effectiveness of Alternative Screening Strategies in South Africa
Source: PLoS One. 2016 Jul 8;11(7):e0158986. doi: 10.1371/journal.pone.0158986 (PMC4938608; doi:10.1371/journal.pone.0158986)
Supplement: S1 Appendix — (DOCX) [file pone.0158986.s001.docx]

Appendix 1. Treatment cost calculations in detail

| **Preemptive fluconazole treatment costs (see details below)** | Rand 2015 | USD |
| --- | --- | --- |
| Cost of preemptive fluconazole treatment (full adherence) | 455.72 | 30.09 |
| Cost of preemptive fluconazole treatment (partial-adherence) | 274.8 | 18.14 |
|  |  |  |
| **Hospitalization costs (see details below)** |  |  |
| Total in-patient costs per hospitalization if patient survives | 29,535.00 | 1950.1 |
| Total in-patient costs per hospitalization if patient dies | 11,814.00 | 780.04 |
| Cost of maintenance fluconazole treatment (post-hospitalization; full adherence) | 407.84 | 26.93 |
| Cost of maintenance fluconazole treatment (post-hospitalization; non-adherence) | 305.88 | 20.2 |

| **Preemptive fluconazole treatment (details)** | 2015 Master Procurement |  |
| --- | --- | --- |
|  | Rand |  |
| Cost of Fluconazole per Package (28 Tablets, 200 mg) | 23.94 |  |
| Number of tablets per package | 28 |  |
| Cost per 200 mg tablet | 0.86 |  |
| Cost per 400 mgs (2 tablets) | 1.71 |  |
| Cost per 800 mgs (4 tablets) | 3.42 |  |
| Days 800 mgs | 14 |  |
| Days 400 mgs | 56 |  |
| Days 200 mgs | 365 |  |
| Cost of preemptive fluconazole treatment (PFT)), full adherence | 455.72 |  |
| Definition of "non-adherence' (as proportion of full adherence) | 0.6 |  |
| Cost of preemptive fluconazole (non-adherence) | 274.8 |  |

| **Hospitalization costs** |  |  |
| --- | --- | --- |
| In-patient days | 15 |  |
| Patient day equivalent cost per day (Rand) | 1969 | PDE District Health Barometer, SA average |
| Total costs for in-patient care and treatment if patient survives full treatment (Rand) | 29,535.00 |  |
|  |  |  |
| If patient dies in hospital, share of 15 days prior to death | 0.4 |  |
| Total costs of in-patient care and treatment if patient dies in hospital | 11814 |  |
|  |  |  |
| **Cost of maintenance fluconazole treatment (as outpatient)** | 2015 Master Procurement |  |
|  |  |  |
| Cost of Fluconazole per Package (28 Tablets, 200 mg) | 23.94 |  |
| Number of tablets per package | 28 |  |
| Cost per 200 mg tablet | 0.86 |  |
| Cost per 400 mgs (2 tablets) | 1.71 |  |
| Cost per 800 mgs (4 tablets) | 3.42 |  |
| Days 800 mgs | 0 |  |
| Days 400 mgs | 56 |  |
| Days 200 mgs | 365 |  |
| Cost of maintenance fluconazole treatment (post-hospital), full adherence | 407.84 |  |
| Definition of "non-adherence' (as proportion of full adherence) | 0.75 |  |
| Cost of maintenance fluconazole (non-adherence) | 305.88 |  |
|  |  |  |
| Total cost if hospitalized (full hospital care and adherence to maintenance fluconazole) | 29942.84 |  |

| **Alternative for hospital costs based on Jarvis et al. 2013** |  |  |
| --- | --- | --- |
| In-patient care and treatment costs |  |  |
| Hotel 15 days | 2266.03 | USD 2010 |
| 4 full blood counts | 26.59 | USD 2010 |
| 1 liter saline for 14 days | 19.04 | USD 2010 |
| 2 lumbar punctures | 34.08 | USD 2010 |
| 4 other tests (creatinine, electrolyte, urea) | 53.02 | USD 2010 |
| 2 additional outpatient visits | 51.74 | USD 2010 |
| total USD 2010 | 2450.5 | USD 2010 |
| Convert to Rand 2010 (exchange rate of 7.34 Rand/$) | 17986.67 | Rand 2010 |
| Inflate to 2014 Rand | 23166.15 | Rand 2014 |
| **Hotel costs as share of total hospital costs** | 0.9247 |  |
